# Ekbom syndrome: a case report

**DOI:** 10.1192/j.eurpsy.2022.2045

**Published:** 2022-09-01

**Authors:** D. Bosnjak Kuharic, M. Cvitanic Mazuran, D. Polšek, M. Herceg, A. Jambrosic Sakoman

**Affiliations:** University Psychiatric Hospital Vrapče, Department For Psychotic Disorders, Zagreb, Croatia

**Keywords:** Delusional disorder, ekbom syndrome, parasitosis, dermatology

## Abstract

**Introduction:**

Ekbom syndrome is a clinical term for delusional parasitosis, a condition characterized by the belief that one’s skin is infested by invisible parasites. Despite having no medical evidence, patients strive to prove their illness and interpret different sensations and symptoms as infestation with parasites.

**Objectives:**

Our objective was to present a case report of a patient with Ekbom syndrome with detailed clinical information and treatment complications.

**Methods:**

We included patient’s history, psychiatric evaluation, complete diagnostic work-up, therapy and follow-up.

**Results:**

A 60-years old female patient was admitted to her first hospital treatment in our psychiatric clinic. Upon admittance, she was extremely tense, preoccupied with the idea that bed bugs have infested her body. She showed extensive medical documentation, including numerous dermatologic reports regarding her condition, interpreting them in accordance with her delusions. In attempt to help herself and “release” the bugs, she harmed herself causing multiple skin lesions across her body and face. The treatment was complicated with secondary skin infections, ulcers, cellulitis and oedemas. Initial treatment with olanzapine was switched to risperidone due to side-effects (sedation, increase of appetite, weight gain). Gradually, with pharmacological treatment, psychoeducation and support, remission was achieved, but poor insight to her previous condition and psychiatric symptoms remained.

**Conclusions:**

Ekbom syndrome presents a serious disorder that can be complicated with secondary somatic complications, often requiring involvement of different medical specialists. Moreover, lack of insight into the need for psychiatric treatment can lead to therapy discontinuation and relapse of symptoms.

**Disclosure:**

No significant relationships.

